# Annual acknowledgement of reviewers

**DOI:** 10.1186/1755-8794-7-6

**Published:** 2014-02-07

**Authors:** Timothy R Sands

**Affiliations:** 1 BioMed Central, Floor 6, 236 Gray's Inn Road, London WC1X 8HB, UK

## Abstract

**Contributing reviewers:**

The editors of BMC Medical Genomics would like to thank all our reviewers who have contributed to the journal in Volume 6 (2013).

## 

Michael Abend

Germany

Sharon Adams

USA

Zubair Ahmed

USA

Eva Albrecht

Germany

Micheala Aldred

USA

Reid Alisch

USA

Rami Aqeilan

Israel

Joel Bader

USA

Vladimir Bajic

Saudi Arabia

Julia Barthold

USA

Brian Bennett

USA

Antoni Berenguer-Llergo

Spain

Francesco Bertoni

Switzerland

Fay Betsou

Luxembourg

Usama Bilal

Spain

Douglas Bittel

USA

Rehannah Borup

Denmark

Paul Boutros

Canada

Peter Kamp Busk

Denmark

Jean Cadigan

USA

Sebastiano Calandra

Italy

Christopher Cardinale

USA

Paloma Cejas

Spain

Jeffrey Chang

USA

Yangchao Chen

Hong Kong

Chao Cheng

USA

I-Fang Chung

Taiwan

Kwang Chul Chung

South Korea

Miguel Coca-Prados

USA

Lee Cooper

USA

Qinghua Cui

China

Josef Davidsson

Sweden

Alessandro De Luca

Italy

Luiz De Marco

Brazil

Johan den Dunnen

Netherlands

Diego di Bernardo

Italy

Chunming Ding

Singapore

Sorin Draghici

USA

Jiajun Du

China

Ranhui Duan

China

Shiwei Duan

China

Aaron Dumont

USA

Ken Dutton-Regester

Australia

Serenella Eppenberger-Castori

Switzerland

Francis (Biao) Feng

Canada

Xin Feng

USA

Patrick Fenichel

France

Elisabetta Fratta

Italy

Ian Gallicano

USA

Niladri Ganguly

UK

Daniel Gatti

USA

Cicek Gercel-Taylor

USA

Stephen Glatt

USA

Ivan Gorlov

USA

Mireia Guerau-de-Arellano

USA

Dominique Guerrot

France

Andre Guillouzo

France

Junming Guo

China

Yong Guo

China

Balazs Györffy

Hungary

Ke Hao

USA

Jennifer Harris

Norway

Florentine Hilbers

Netherlands

Andrew Horvai

USA

Peng Hou

China

Pei-Yin Hsu

USA

Tinghui Hu

USA

Chi-Ying Huang

Taiwan

Rita Humeniuk

USA

Yong-Hui Jiang

USA

Glen Jickling

USA

Wenjun Ju

USA

Zheng Junch

China

Adetayo Kasim

UK

Chiea Chuen Khor

Singapore

Il-Jin Kim

USA

Benjamin Kipp

USA

Tohru Kiyono

Japan

Ulf Klein

USA

Jean-Pierre Kocher

USA

György Kosztolányi

Hungary

Pawel Krawczyk

Poland

Shunichiro Kubota

Japan

Thomas LaFramboise

USA

Xun Lan

USA

Ann Lee

Singapore

Ju-Seog Lee

USA

Ulrich Lehmann

Germany

Jia-Da Li

China

Unhee Lim

USA

Curt Lind

USA

Charlotte Ling

Sweden

Chunyu Liu

USA

Da-Zhi Liu

USA

Qi Liu

USA

William Lockwood

USA

Steven Mack

USA

Lars Maegdefessel

Sweden

Giancarlo Marra

Switzerland

Zubin Master

USA

Benjamin Meder

Germany

Lorenzo Melchor

UK

Lourdes Mengual

Spain

Fredrik Mertens

Sweden

Rongjuan Mi

USA

Yutaka Midorikawa

Japan

Pablo Minguez

Germany

Katherine Mitsouras

USA

Yuji Miyazaki

Japan

Richard Moser

USA

Mette Myklebust

Norway

Denise Nishita

USA

Yury Nunez

USA

Michael O'Connell

USA

Jun Ohashi

Japan

Yukinori Okada

USA

Fabiola Olivieri

Italy

Ana Osorio

Spain

Fumitaka Oyama

Japan

Hatice Gulcin Ozer

USA

Helen Pan

USA

Jong Park

USA

Yongseok Park

USA

Domingo Pascual-Figal

Spain

Paul Perco

Austria

Ana Petelin

Slovenia

Laila Poisson

USA

Toni Pollin

USA

Shuhong Qiao

USA

Jiannis Ragoussis

UK

Evica Rajcan-Separovic

Canada

Olaf Riess

Germany

Miguel Rivera

Spain

Helen Rizos

Australia

Pedro Pereira Rodrigues

Portugal

Raul Rodriguez-Esteban

Switzerland

Cristina Romei

Italy

Iwona Rudkowska

Canada

Ivan Rusyn

USA

George Sachs

USA

Hiroyuki Sakai

Japan

Lakshman Samaranayake

Hong Kong

Ian Sayers

UK

Aneta Schaap-Oziemlak

Netherlands

Rodney Scott

Australia

David Serre

USA

Antony Shrimpton

USA

Xueling Sim

USA

Andrew Sims

UK

Ann Smeets

Belgium

Di Smith

USA

Enrico Solcia

Italy

Ken Song

USA

Maud Starmans

Canada

Renske Steenbergen

Netherlands

Mark Stoneking

Germany

Manikkam Suthanthiran

USA

Hiromu Suzuki

Japan

Maurizio Taglialatela

Italy

Aik Choon Tan

USA

Daniel Tan

Singapore

Eng Choon Tan

Singapore

Patrick Tan

Singapore

Carl Troein

Sweden

George Tseng

USA

Steven Van Laere

Belgium

Wessel van Wieringen

Netherlands

Emily Vucic

Canada

William Wade

UK

Lin Wan

USA

Hsei-Wei Wang

Taiwan

Shengyue Wang

China

Xiaowei Wang

USA

Peter White

USA

Nedra Whitehead

USA

Janey Wiggs

USA

Yaguang Xi

USA

Zhousheng Xiao

USA

Yoshihisa Yamashita

Japan

Weichuan Yu

Hong Kong

Katherine Yutzey

USA

Tanja Zeller

Germany

Xianquan Zhan

China

Jie Zhang

USA

Sheng Zhang

USA

Zhiguo Zhang

USA

Hua-Chuan Zheng

China

Elias Zintzaras

Greece

## Pre-publication history

The pre-publication history for this paper can be accessed here:

http://www.biomedcentral.com/1755-8794/7/6/prepub

